# Loss of capillary pericytes and the blood–brain barrier in white matter in poststroke and vascular dementias and Alzheimer’s disease

**DOI:** 10.1111/bpa.12888

**Published:** 2020-08-14

**Authors:** Ren Ding, Yoshiki Hase, Kamar E. Ameen‐Ali, Michael Ndung'u, William Stevenson, Joseph Barsby, Ryan Gourlay, Tolulope Akinyemi, Rufus Akinyemi, Maiko T. Uemura, Tuomo Polvikoski, Elizabeta Mukaetova‐Ladinska, Masafumi Ihara, Raj N. Kalaria

**Affiliations:** ^1^ Neurovascular Research Group Translational and Clinical Research Institute Newcastle University Campus for Ageing & Vitality Newcastle Upon Tyne UK; ^2^ Institute for Advanced Medical Research and Training College of Medicine University of Ibadan Ibadan Nigeria; ^3^ Institute on Aging and Center for Neurodegenerative Disease Research Department of Pathology and Laboratory Medicine Perelman School of Medicine University of Pennsylvania Philadelphia PA; ^4^ Department of Neuroscience, Psychology and Behaviour University of Leicester Leicester UK; ^5^ Department of Neurology National Cerebral and Cardiovascular Center Osaka Japan

**Keywords:** cerebral capillary, collagen type IV, dementia, pericytes, platelet‐derived growth factor receptor, vascular dementia, white matter

## Abstract

White matter (WM) disease is associated with disruption of the gliovascular unit, which involves breach of the blood–brain barrier (BBB). We quantified pericytes as components of the gliovascular unit and assessed their status in vascular and other common dementias. Immunohistochemical and immunofluorescent methods were developed to assess the distribution and quantification of pericytes connected to the frontal lobe WM capillaries. Pericytes with a nucleus were identified by collagen 4 (COL4) and platelet‐derived growth factor receptor‐β (PDGFR‐β) antibodies with further verification using PDGFR‐β‐specific ELISA. We evaluated a total of 124 post‐mortem brains from subjects with post‐stroke dementia (PSD), vascular dementia (VaD), Alzheimer’s disease (AD), AD‐VaD (Mixed) and post‐stroke non‐demented (PSND) stroke survivors as well as normal aging controls. COL4 and PDGFR‐β reactive pericytes adopted the characteristic “crescent” or nodule‐like shapes around capillary walls. We estimated densities of pericyte somata to be 225 ±38 and 200 ±13 (SEM) per COL4 mm^2^ area or 2.0 ± 0.1 and 1.7 ± 0.1 per mm capillary length in young and older aging controls. Remarkably, WM pericytes were reduced by ~35%–45% in the frontal lobe of PSD, VaD, Mixed and AD subjects compared to PSND and controls subjects (*P* < 0.001). We also found pericyte numbers were correlated with PDGFR‐β reactivity in the WM. Our results first demonstrate a reliable method to quantify COL4‐positive pericytes and then, indicate that deep WM pericytes are decreased across different dementias including PSD, VaD, Mixed and AD. Our findings suggest that downregulation of pericytes is associated with the disruption of the BBB in the deep WM in several aging‐related dementias.

## Introduction

Older age is identified as the single most important risk factor contributing to increased white matter hyperintensities (WMH) on T2‐weighted magnetic resonance imaging. Increased volumes of WMHs are associated with vascular disease, disability, cognitive impairment and death (17, 24, 34, 41). WMHs have been largely attributed to cerebral small vessel disease (SVD) but large vessel disease may also contribute to WM lesions (5, 17, 32). We have previously identified the cell and molecular changes within the gliovascular unit that incorporates the blood–brain barrier (BBB) in the deep frontal WM of elderly patients who develop dementia after stroke (13, 30). Perivascular astrocytic degeneration characterized by clasmatodendrosis was a key feature associated with stroke survivors who developed dementia relative to those who remained cognitively intact. However, it is unknown how other key cellular components of the gliovascular unit such as pericytes are affected in stroke survivors or those who develop vascular dementia (VaD).

Pericytes function like mural cells on the capillary endothelium, on precapillary arterioles and on postcapillary venules (7, 9, 10, 20). Multiple functions beyond contractile control of blood flow (26) are attributed to pericytes including paracrine signaling during angiogenesis and phagocytosis. Pericytes play an important role in BBB function (53). Pericyte coverage on brain capillaries in the mouse at least is estimated to be >90% (9) that is characterized by longitudinal and circumferential processes around capillaries. The platelet‐derived growth factor (PDGF) is one of the key signaling pathways identified in pericytes (38). Endothelial PDGF‐β recruits pericytes via the PDGF receptor‐β (PDGFR‐β) and disruption of PDGFR‐β signaling results in fewer recruited pericytes to the vessel, causing vessel leakage, tortuosity, microaneurysms and microbleeds (12). In this context, we showed that bone morphogenetic protein‐4 (BMP4) was highly expressed in WM pericytes in cerebral SVD (52). Chronic cerebral hypoperfusion upregulated BMP4 to promote angiogenesis but induced astrogliogenesis at the expense of oligodendrocyte precursor cell proliferation and maturation, thereby aggravating WM damage.

The lack of reliable markers of pericytes had hampered their complete characterization (50). A reliable, robust method to identify and quantify pericytes would accelerate the research and deepen our understanding on the role of pericyte in brain disorders. In this study, we used a simple reliable method to identify pericytes and determined their status across different neurocognitive disorders including poststroke dementia (PSD), VaD, Alzheimer’s disease (AD) and mixed dementia with vascular and Alzheimer pathologies with the hypothesis that they are vulnerable in diffuse WM disease, which is one of the signatures of SVD. In addition, we evaluated perfused post‐mortem brain tissue from nonhuman primates subjected to cerebral hypoperfusion (13). We focused on pericytes because of their close relationship with endothelial cells (29) and they are key cellular components of the gliovascular unit, which disintegrates during WM damage. We concentrated on the frontal lobe because of its contiguity with the centrum semiovale region and its relative vulnerability in cerebrovascular diseases (35, 36).

## Materials and methods

### Subjects

Table 1 provides demographic details and diagnoses in 124 subjects derived from our longitudinal prospective dementia series and aging controls. Dementia was clinically diagnosed and pathologically confirmed by post‐mortem examination as either AD, mixed AD‐VaD, (Mixed), VaD or PSD. Available radiological reports indicated WM changes consistent with SVD of the brain in most dementia patients (31). In addition, we assessed poststroke no dementia (PSND) subjects as well as young and older controls. The mean age of the older controls was not different from any of the dementia subjects. The controls, aged 55–99 years were participants either in previous prospective studies or from unrelated brain donations to the Newcastle Brain Tissue Resource (NBTR). They were selected to be included as controls if they had no cognitive impairment or clinical or pathological evidence of neurological or psychiatric disease. The VaD, PSD and PSND subjects were from the Newcastle Cognitive Function after Stroke study (2). Local research ethics committees of the Newcastle upon Tyne NHS Foundation Hospitals Trust granted ethical approvals. Permission to use brains for post‐mortem research was also granted by consent from the individuals themselves when they had been still alive or next‐of‐kin or family member. All brain tissues were obtained from the NBTR.

**Table 1:** Demographic details of all the cases and controls.

Variable	Young controls	Old controls	PSND	PSD	VaD	Mixed	AD
N	12	20	21	20	17	18	16
Mean age, years (range)	57.5 (46–65)	79.3 (78–94)	85.1 (75–96)	87.1 (75–96)	84.2 (71–98)	85.1 (72–93)	84.2 (76–96)
Gender (M:F %)	55:45	35:65	57:43	30:70	41:59	44:56	56:44
MMSE, mean ± SEM	na	29 ± 1	27 ± 0.4	16 ± 1	13 ± 4	11 ± 2	7 ± 2
CAMCOG, mean ± SEM	na	na	90 ± 1	66 ± 3	na	na	39 ± 7
Braak stage, mean (range)	0.25 (0–1)	1.9 (0–4)	2.6 (1–4)	2.6 (1–4)	2.0 (0–4)	5.2 (5–6)‡	5.6 (5–6)‡
CERAD, mean (range)	0.0 (0–0)	0.5 (0–2)	1.7 (1–2)	1.3 (1–3)	1.0 (0–2)	2.9 (2–3) ‡	2.9 (2–3) ‡
ABC scores, mean	na	A0.5, B1.2, C0.5	A0.5, B1.2, C0.7	A0.5, B1.2, C0.8	A0.6, B1.2, C0.8	A2.5, B2.6, C2.6	A3, B3, C3
CAA frequency (moderate‐severe), %	0	6	15	18	17	9	39
Vascular pathology score, mean (range)^†^	na	6.7 (0–10)^†^	13.5 (13–14)	13.3 (9–17)	13.2 (10–16)	11.0 (6–14)	10.8 (3–16)
WML score, mean (range)	na	0.5 (0–2)^†^	2.5 (2–3)	2.4 (2–3)	2.9 (2–3)	2.9 (2–3)	1.8 (0–3)
White matter/vascular lesions, moderate‐severe (%)	na	17.6**	100	100	100	95	72

Numbers represent mean values (±SEM) and where given with the range of values in parentheses. The causes of death included bronchopneumonia (95%), cardiac arrest and carcinoma, renal failure and gastrointestinal bleed with no particular distribution pattern in any group. The post‐mortem interval between death and tissue retrieval ranged 24–47 h for all the cases. There were no differences in the length of post‐mortem delay between groups. Braak staging scores and Alzheimer’s disease neuropathologic changes (45) were different in mixed and AD cases compared to all other groups (*P* < 0.05). Mean vascular pathology scores (range) for PSND and PSD groups were 13.5 (13–14) and 13.3 (9–17) compared to 6.7 (0–10) for controls (^†^ *P* < 0.05). These scores were derived as described previously (18). WML Score, white matter pathology score assessed using scale from (18). Mean WML Score was high in all poststroke and dementia subjects compared to controls (^†^ *P* < 0.01). WM/Vascular lesions, **P < 0.01 compared to all poststroke and dementia subjects.

Abbreviations: ABC = AD Neuropathology scoring system; AD = Alzheimer’s disease; CAA = cerebral amyloid angiopathy; CAMCOG = Cambridge cognition examination; F = female; M = male; MMSE = Mini Mental state examination; N = number of subjects; na = not available; NPD = no pathological diagnosis; PSND = poststroke non‐demented; PSD = poststroke dementia; VaD = vascular dementia; WM = white matter.

### Brain tissues and neuropathological analyses

Neuropathological assessment was carried out as described previously (32). Briefly, hematoxylin and eosin (H&E) staining was used for assessment of structural integrity and infarcts, Nissl and Luxol Fast blue staining for cellular patterns and myelin loss, Bielschowsky’s silver method and amyloid‐β immunohistochemistry for ABC rating of neuritic plaques, Gallays stain for neuritic pathology and tau immunohistochemistry for Braak staging of neurofibrillary tangles. The clinical diagnosis of AD was confirmed on evidence of significant Alzheimer’s‐type pathology incorporating Braak stages V–VI, moderate‐severe CERAD (40) and high ABC scores per National Institute of Aging‐Alzheimer’s Association guidelines (45) and an absence of significant vascular pathology. The clinical diagnosis of VaD was made when there were multiple or cystic infarcts, lacunae, border‐zone infarcts, microinfarcts and SVD and pathologically confirmed as Braak stage ≤ IV (36, 37). Mixed AD and VaD case was classified when there was sufficient degree of AD pathology (45) and significant vascular pathology (Table 1).

Vascular pathology scores were derived from the presence of vascular lesions/pathologies as described previously (18). WM lesion (WML) scores were determined on scale of 0 to 3 signifying none, mild, moderate and severe. Previously, we had shown there was 95% agreement in scoring between two assessors (18). WM/vascular lesion severity was graded from low to severe in quartiles essentially as described previously (33). All the vascular measures were compatible with the recently established vascular cognitive impairment neuropathology consortium criteria (49). Tissues from control subjects had occasional aging‐related pathology and were classified as “no pathological diagnosis” (Table 1). Except for neuropathological examination (RNK), all subsequent morphological analyses were undertaken under operator‐blinded conditions. Samples were identified with coded sequential numbers. In addition, at least two of both positive and negative controls were included to monitor the quality of staining.

### Immunohistochemistry methods

Formalin‐fixed paraffin‐embedded whole coronal sections at levels 6–8 (36, 48) containing the frontal lobe (Brodmann area 9) were analyzed. When sampling tissue, we ensured to select the WM regions free of any obvious infarcts and thus assessed effects of remote stroke injury. Immunohistochemistry was performed to examine various microvascular structures essentially as described before (16, 29). The following antibodies were used to assess various cellular features: collagen IV (COL4 at dilution 1:1000, C1926, Merck (Sigma‐Aldrich), Branchburg, NJ, USA), a marker of basement membrane in the vessels, platelet‐derived growth factor receptor‐β (PDGFR‐β at 1:200 dilution, clone 42G12, #AF385, R&D systems, Minneapolis, MN, USA), a marker for pericytes, bone morphogenetic protein 4 (BMP4 dilution at 1:100, MBA1049, Millipore, MA, USA), α‐smooth muscle actin (αSMA at dilution 1:1000, Clone 1A4, Dako, Cambridge, UK), a marker for mural cells and glucose transporter‐1 (GLUT‐1 at 1:200, PA1‐21041, Fisher Scientific, Waltham, MA, USA), a marker of endothelial cells. Vectastain ABC mouse kits (PK‐6102, Vector Laboratories, Burlingame, CA, USA) and DAB were used to localize single or double immunohistochemical stains. Tissue sections were then counter stained with hematoxylin to visualize the landmarks.

### Electron microscopy

Post‐mortem brain tissues from relevant controls were sampled from the gray matter and WM for the temporal lobe. Subsequent to 16‐h fixation in 2% paraformaldehyde and 0.05% glutaraldehyde in 0.1 M phosphate buffer (PB, pH7.4), samples were rinsed in two changes of PBS. Half of each batch of samples was treated with 1% osmium tetroxide (OsO4) in 0.1 M PB. Samples were dehydrated in increasing concentrations of alcohol, cleared in propylene oxide and then, embedded in epoxy resin. Sections were cut into 1 μm using an Ultracut microtome (Reichert‐Jung, Depew, NY) and stained with 1% Toluidine blue and 1% borax in order to confirm the position of blood vessels in the sections. Ultrathin sections were cut using a DDK Delaware diamond knife and placed onto 3.05 mm nickel 300 square mesh grids, which had been pre‐treated with 10% nitric acid. OsO4 treated sections were used for morphological examination (57).

### Immunofluorescence methods

Tissue sections were first treated with 0.1 mg/mL protease and then, incubated overnight at 4°C with primary antibodies to anti‐COL4 (C1926 Sigma) monoclonal antibody, anti‐PDGFR‐β, (1:200 dilution, AF385, R&D Systems), αSMA (1:500 dilution, Clone 1A4, Dako) and glucose transporter‐1 (GLUT‐1, 1:200, Thermo Scientific). Sections were washed with PBS and further incubated with donkey anti‐goat conjugated Alexa Fluor 594 (1:1000, A11058, Thermo Fisher Scientific, Waltham, MA, USA) and rabbit anti‐mouse Alexa Fluor 488 (1:1000, A11059, Thermo Fisher Scientific). Sections were then washed in PBS before mounting in Vectashield with DAPI (H‐1200, Vector Laboratories). Images were captured using a Leica TCS SP2 (upright) and Zeiss Spinning Disk (Invert) confocal microscopes as described previously (15).

### COL4 specific histochemistry to assess pericytes

**Figure 1:**
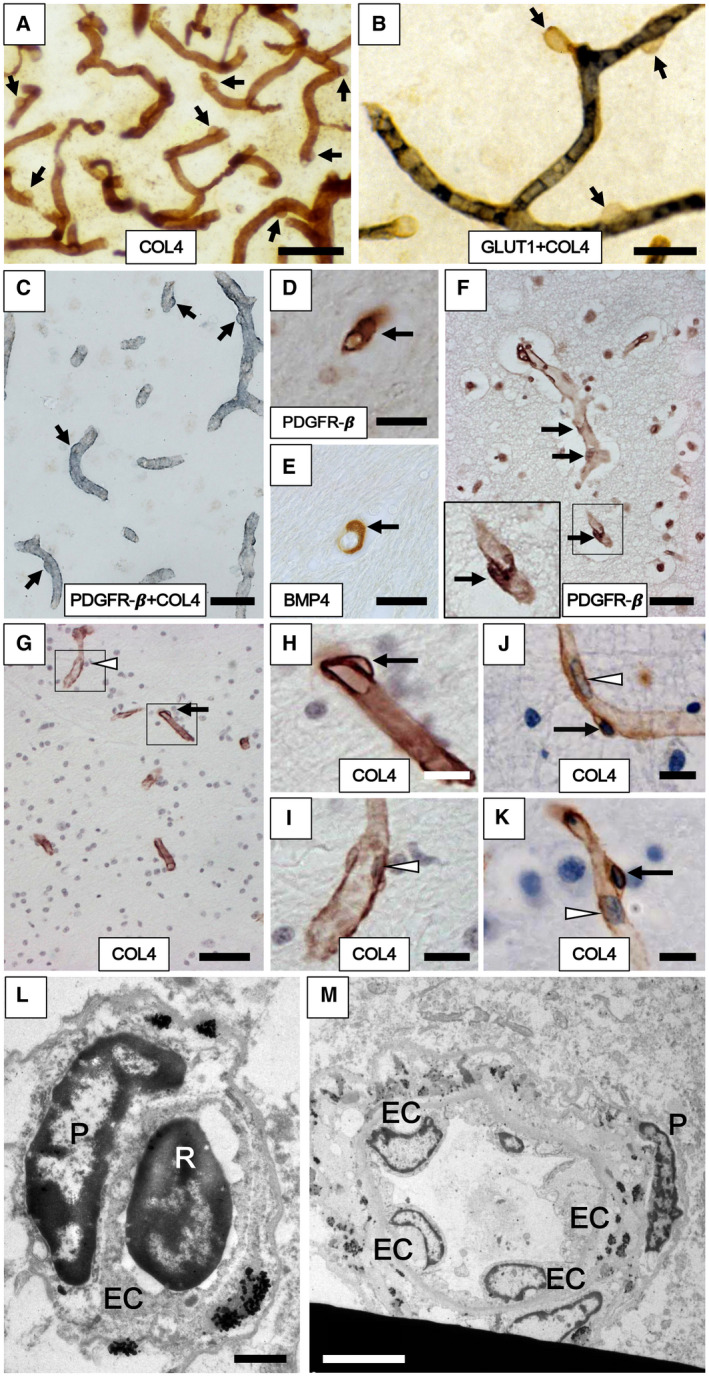
*Cerebral capillaries with pericytes in the frontal cortex and WM of aging subjects*. **A–F.** Pericytes evident as “bumps” or “crescent” shaped cells in the capillary network immunostained with antibodies to COL4 (**A, G, H, I, J** and **K**), GLUT1 (blue‐black) and COL4 (brown) (**B**), PDGFR‐β (brown) and COL4 (blue/gray) (**C**), PDGFR‐β (**D, F**) and BMP4 (**E**). Arrows show location of the pericyte cell bodies along the capillaries. **G–K**. Pericyte soma (black arrows in **G, H, J** and **K**) and endothelial cell (white arrowhead in **G** and **I**) and other types of cells including erythrocytes observed within the lumen (white arrowhead in **J** and **K**) in the frontal WM capillaries of the Baboon (**G**–**I**) and human (**J** and **K**). **L,M.** Transmission electron microscope images show pericyte cell body (**P**) on a WM capillary (**L**) and precapillary/small arteriole (**M**) in cross‐section from 65‐year‐old male subject. The marked differential localization between the pericyte and endothelial cell (EC) and the lumen with an erythrocyte (R for red blood cell) can be noted. **L–M** also assure assessment of non‐capillary pericytes or EC or smooth muscle cell nuclei were not included in the pericyte counts. Images in **A and B** were from 20 µm thick sections, whereas those from **C** to **I** were from 10 µm sections. Length density of frontal cortical capillaries (**A**) was greater than WM (**B**). Magnification bars: A, G = 50 µm; C, F = 30 µm; B, D, E = 20 µm; H, I, J, K = 10 µm; L = 1 µm; M = 5 µm.

**Figure 2:**
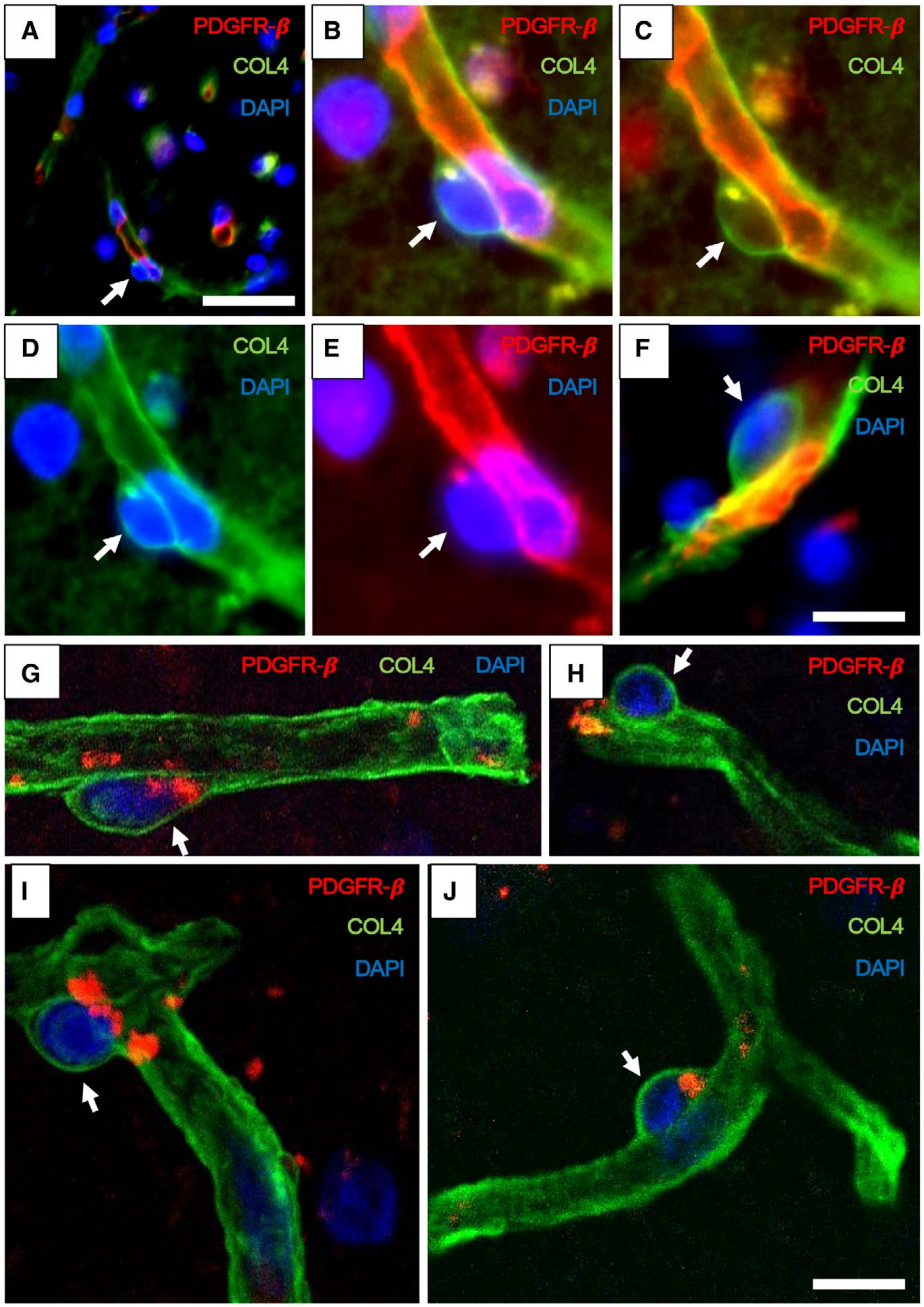
*Pericyte soma on WM capillaries demonstrated by immunofluorescence*. **A–F.** PDGFR‐β (red) and COL4 (green) immunofluorescence staining with nuclei (DAPI) (blue). **A,B.** Low‐ and high‐power images showing capillary segments (arrowheads) with overlapping PDGFR‐β (red), COL4 (green) and DAPI (blue). **C.** Same vessel segment as B with PDGFR‐β (red) and COL4 (green); **D.** COL4 (green) and DAPI (blue); **E.** PDGFR‐β (red) and DAPI (blue). **F.** Another capillary segment with PDGFR‐β (red), COL4 (green) and DAPI (blue) clearly showing pericyte cell body. **G–J.** Images taken by a confocal microscope showing pericyte cell bodies (arrows) with nuclear stain (DAPI). Capillaries and pericyte processes are revealed by COL4 and PDGFR‐β (red) immunoreactivities. Magnification bars: A = 50 µm, F, J = 10 µm.

How to reliably identify pericytes has been a key question in microvascular research. In view of the general absence of a single marker of pericytes for robust use in human tissues, we developed COL4 immunohistochemistry as a readily applied method to determine densities of microvascular pericytes in disease and in experimental animals (Figures 1 and 2). As described above, tissue sections were immunostained with well‐characterised COL4 antibodies and then, counterstained with hematoxylin (Figure 1). Pericytes with a nucleus characteristically recognized as nodules or “bumps” or “crescent” shaped cell bodies were counted manually along capillary profiles from more than 2000 captured images. The total number of pericyte cell bodies were then determined for each case from 8 to 25 frames per case and then, a mean number was calculated per case. In total, we counted over 2100 pericytes involving 1450 images with each group comprising at least 10 cases. A pericyte (cell body) was counted only if it had the characteristic shape and there was a clear nucleus within as identified by hematoxylin under ×40 magnification. We were careful to count pericytes wrapped round COL4 on abluminal surface of the capillary only, unlike luminally localized endothelial cells (Figure 1A–K). In contrast to previous studies (44), we did not determine pericyte cell coverage because our intent was to assess potential change in pericyte nuclei as a more accurate measure of pericyte status in chronic disorders. Throughout, the histopathological analyses were performed blind to the operator. The inter‐rater reliability in recognizing COL4 positive pericyte soma by two independent investigators (RD and JB) was more than 95% with the kappa score of 0.96.

### Image acquisition and analysis of pericyte densities

**Figure 3:**
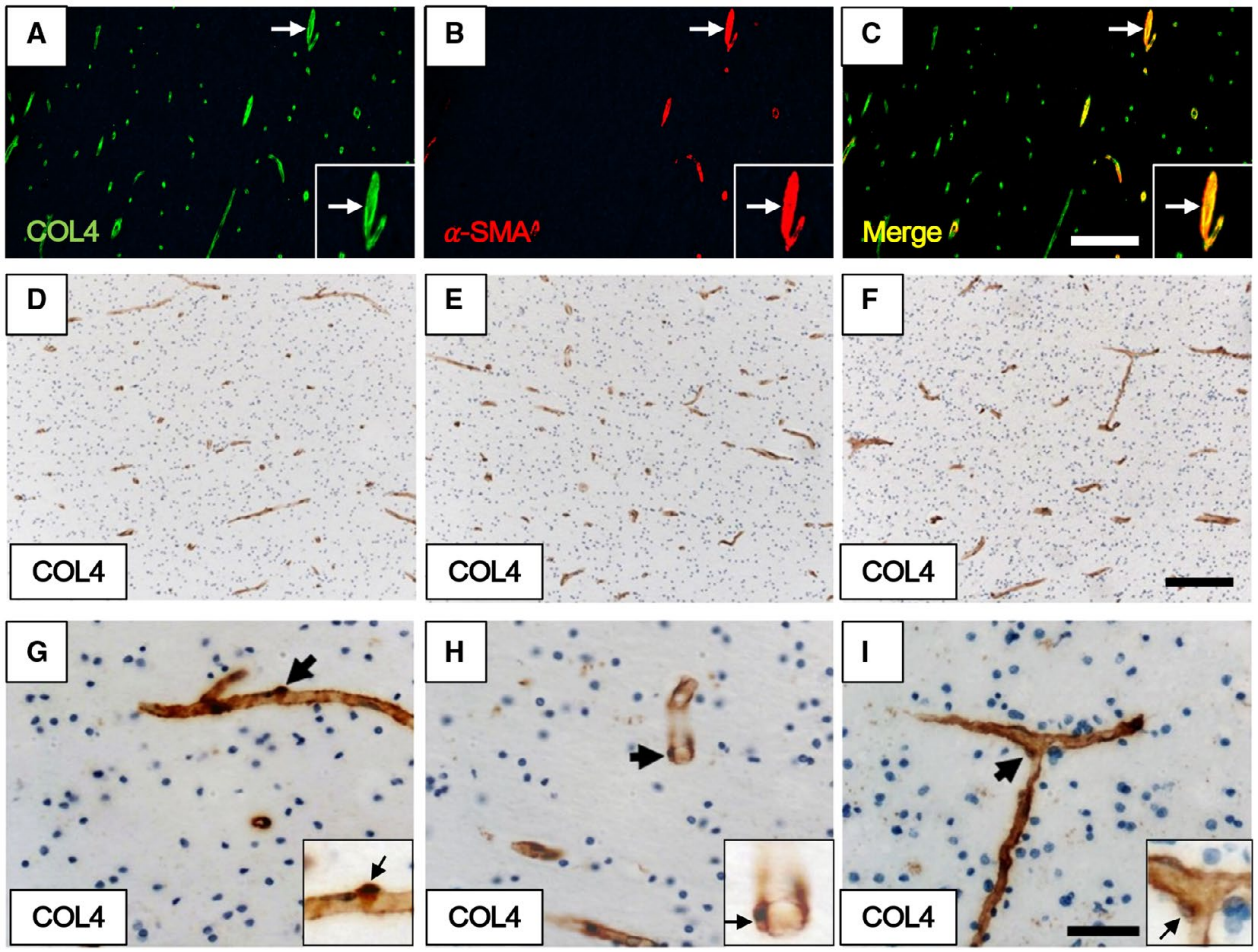
*Quantification methods used for determining density of pericytes associated with capillaries*. **A–C.** Immunofluorescent staining of WM capillaries and arterioles. **A.** COL4 (green). **B.** α‐SMA (red). **C.** merged image of **A** and **B**. Quantification of pericytes was performed on COL4‐positive but α‐SMA‐negative capillary profiles. Each inset showing an arteriole double‐positive for COL4 and α‐SMA (white arrow) at higher magnification. **D–I.** Pericyte cell bodies revealed by COL4 immunostaining and hematoxylin nuclear counterstain as crescent shapes or as bumps on capillaries of the WM. Pericytes cell bodies are evident in longitudinal (**G**) and transverse profiles (**H**) as well as at intersections (**I**) of capillaries. **G–I.** Each inset showing a pericyte (arrow) at higher power. Magnification bars: C, F = 200 µm; I = 50 µm.

Images of capillary beds or regions of interest (ROI) within the tissue sections were captured on a Zeiss Axioplan 2.0 microscope and Image capture software (Infinity Capture V4.6.0, Lumenera Corporation), taking care to avoid large vessels including arterioles >20 µm external diameter (Figure 3D–I). Immunohistochemical staining was quantified using Image‐Pro Plus (V.6.3; Media Cybernetics, Silver Spring, MD, USA). We assessed the percent area (% Area) for each case from at least 10 ROI images representing the vascular area stained with COL4 (as % COL4 Area) and to test the quality of the immunoreactvities between individual sections and cases, we ascertained the integrated optical density (IOD). There were no significant differences in IOD values between disease and control samples. We also found there was a lack of relationship between the immunohistochemical staining of COL4 or PDGFR‐β and length of fixation, or post‐mortem interval, between the groups. The percentage of COL4 stained area (%) and COL4 stained area (mm^2^) were analyzed manually using the Image‐Pro Plus Analyzer. COL4 stained area in mm^2^ was derived using the equation 1% COL4 stained area= 8.0 × 10^−3^ mm^2^.

For the values of pericyte numbers per COL4 area, we divided the numbers of cells by the COL4 area (mm^2^) in each image and calculated a mean value for each case from the number of images. To obtain pericyte soma per unit capillary length, we calculated the total length of capillaries per image and used that value as the denominator to obtain a mean value for each case. Taking into account that human brain capillaries are 5–9 µm in diameter, capillary length was determined from more than 2000 2D images as described previously (11, 29).

### Enzyme linked immunosorbent assays (ELISA)

Protein extracts of frozen tissue from the frontal WM for 10–16 cases in each group were assayed to determine fibrinogen and PDGFR‐β immunoreactivities. Homogenates of tissue were prepared essentially as described previously for the multiplex cytokine assays (14). Fibrinogen was determined using a sandwich ELISA (25). PDGFR‐β was assayed as specified by the manufacturer (Human Total PDGFR beta DuoSet IC ELISA, DYC385, R&D Systems, Minneapolis, MN). For both proteins, the relative values were calculated and units expressed per mg protein.

### Three vessel occlusion model and pericyte analysis in WM of nonhuman primates

Postmortem brain tissues were obtained from adult baboons (*Papio Anubis*) weighing 16–20 Kg (7–12 years old), housed at the Institute of Primate Research (IPR), National Museums of Kenya. The IPR internal review board of the National Museums granted ethical approval and permission for this entire study. As described previously (13), the animals were subjected to permanent occlusion of both the internal carotid arteries and a left vertebral artery for survival periods of 1, 3, 7, 14, 21 and 28 days, as well as sham operated animals (n = 4–8 each group). We evaluated densities of capillary endothelium using H&E and COL4 immunohistochemistry as described above, and the BBB leakage with fibrinogen (1:2000 dilution, A0080, Dako, Cambridge, UK) (13). Pericytes in the deep WM were determined in coronal sections at the level of the frontal lobe in the same manner as described above in human brains. Percentage of fibrinogen stained area (%) in the WM was calculated to evaluate BBB leakage.

### Statistical analyses

Data were analyzed using GraphPad Prism and SPSS (V19.0, IBM). Data were confirmed for normality using the Shapiro–Wilk test. Differences between means of groups were first tested using one‐way ANOVA followed by Tukey’s post hoc test or Kruskal–Wallis H test where appropriate. Linear correlations between PDGFR‐β immunoreactivity and number of pericytes per COL4 area (mm^2^) or per unit capillary length and between fibrinogen and pericyte numbers per COL4 area (mm^2^) were performed using the Pearson’s correlation, as described previously (16). Differences were considered significant with *P*‐value less than 0.05 and data are presented as mean ± SEM.

## RESULTS

### Identification of pericytes in COL4 immunostained capillaries

We had previously noted characteristic nodule‐like “bumps” and “crescent” shaped cell features immunostained with COL4 that were contiguous with the basement membrane of microvessels but were evident as distinct entities and clearly different in size and flat features of luminal endothelial cells (Figure 1). They occurred at irregular intervals in the microvascular network of both the cerebral cortex and WM. They were readily observed along lengths of capillary profiles (~5–9 µm diameter), particularly evident in thicker tissue sections (Figure 1A). They were negative for specific markers of the endothelium such as GLUT1 (Figure 1B). We double immunostained 10 frontal cortex tissue sections with antibodies to GLUT‐1 and COL4 and in none of them was there an overlap between the two markers indicating that COL4+ve cell bodies were not endothelial cells, consistent with our previous work (15). Upon double immunostaining with various cellular markers, we confirmed the “bumps” to be pericytes and positive for PDGFR‐β immunoreactivity (Figure 1C–F). They had similar profiles in both human and baboon WM, which was retrieved from the perfused brains (Figure 1G, cf. F and H). We also found they were positive for BMP4 immunoreactivity (Figure 1E) but these were present only in a proportion of the capillary segments (52). Our observations also confirmed PDGFR‐β immunoreactivity was largely associated with pericytes in capillaries and in the virtual absence of αSMA reactivity (0.1% in 50 capillary profiles). Upon electron microscopy of the post‐mortem tissue, we further confirmed that the large sizes of pericyte cell bodies and their clear differential localization were not those of endothelial cells (Figure 1I–L). As there are pericytes in arterioles or precapillaries, which also have more endothelial cells (Figure 1M), we particularly concentrated on capillary pericytes (<10 µm diameter) associated with BBB.

Using immunofluorescence and confocal microscopy, we further confirmed and validated that the “bumps” and “crescent” like cellular structures were pericyte somata (Figure 2A–F). They were positive for PDGFR‐β, delineated by COL4 immunoreactivity and contained nuclei (Figure 2G–J). We determined the cell somas were ~4–5 µm in diameter (cf. Figure 1H) and frequently observed with extended longitudinal and circumferential processes positive for PDGFR‐β (eg, Figure 2F). Thus, the nodule‐like structures on capillaries immunostained by COL4 were established as bona fide pericyte somata, which were reliably identified for quantification. In parallel experiments, we found that antibodies to laminin, another basement membrane marker could be used to identify pericytes in this manner (data not shown).

In accord with our previous work on the gliovascular unit in the WM in different dementias (30), we focused on the frontal deep WM, which is most frequently afflicted by demyelination and cerebral SVD associated with the VaDs. Quantification of COL4 immunostained pericyte somata on capillaries free of any αSMA (Figure 3A–C) with clear nuclei in the deep WM in normal controls (Figure 3D–I) indicated that the overall mean (±SEM) densities of pericyte cell somas in young controls were estimated to be between 1.8 and 2 ±0.1 mm capillary length. We did not detect the significant differences in pericyte numbers between young and older controls although the mean in older controls was ~15% less (*P* > 0.05, n = 9–10, 200–320 pericytes).

**Figure 4:**
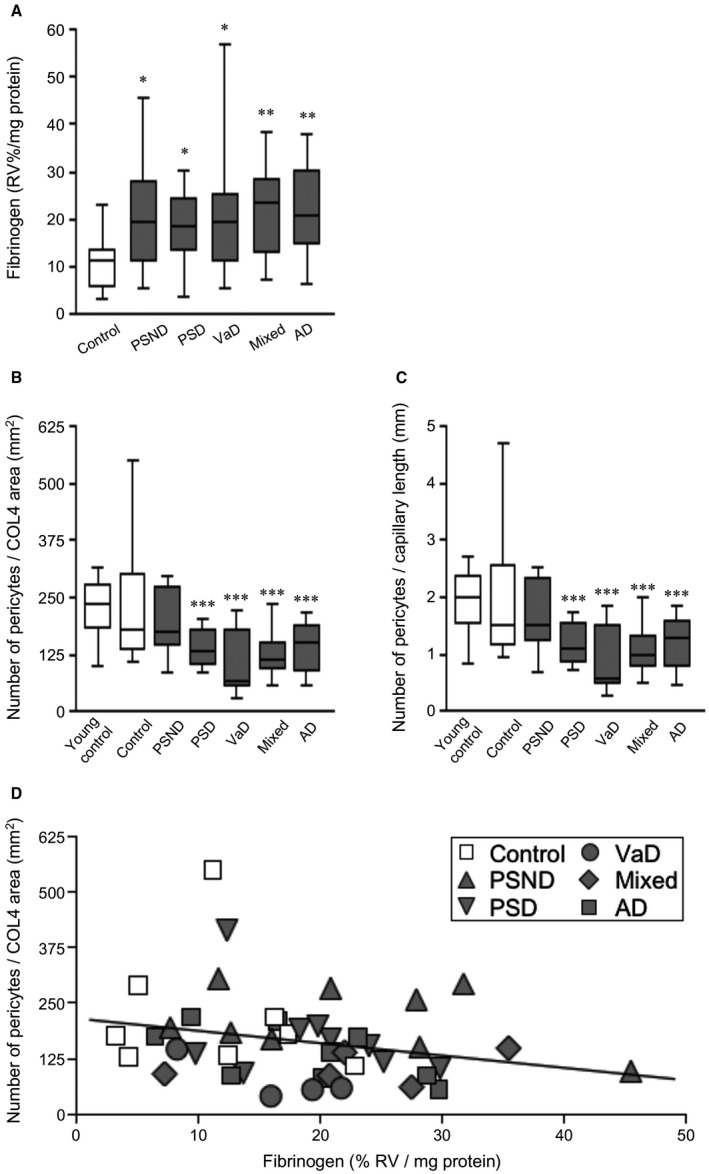
*Quantification of WM pericytes in different dementias compared to normal aging controls*. **A.** Fibrinogen reactivity in frontal WM in control, PSND and different dementia groups. Fibrinogen reactivity measured by ELISA in WM extracts was increased in PSND and all the dementia groups compared to controls (*P < 0.05; **P < 0.01). **B,C.** Box plots show pericyte numbers per COL4 area (mm^2^) (**B**) and per unit (mm) capillary length (**C**). Pericyte numbers were decreased in PSD compared to PSND subjects (***P < 0.001). They were also decreased in all the dementia types compared to controls and young controls. (***P < 0.001, one‐way ANOVA, n = 8–12 cases). Data generated from counts of pericytes per case comprising 110 to 385 cells per each group derived from 82 to 382 separate images. **D.** Correlation of fibrinogen reactivity (% RV, relative values) with pericyte number per COL4 area (mm^2^) (Pearson’s *r* = −0.3, *P* = 0.057). Demographic details of the subjects are given in Table 1.

We next quantified pericyte cell bodies within WM capillaries across several dementias characterized by variable degrees of demyelination and vascular pathology (Figure 4). In tandem with the WM pathology scores in these cases (Table 1), we first ascertained the extent of BBB leakage in the deep WM of subjects with the dementias. Using fibrinogen as a surrogate marker for BBB damage, we found that the specific activity of this extravascular protein was increased in PSD, VaD, Mixed and AD subjects compared to aging controls. However, as expected fibrinogen was also increased in PSND subjects since they exhibit similar vascular pathology to PSD subjects (Figure 4A; Table 1). We found that irrespective of the method used for calculation that is, numbers of pericyte cell bodies per COL4 area (mm^2^) or cell densities per mm capillary length, pericytes were variably decreased by 35%–45% in the frontal WM across all the dementias (Figure 4B,C). Remarkably, VaD subjects were the most affected (Figure 4B). More importantly though, we noted that pericyte numbers in the PSD group were significantly reduced compared to similar age PSND subjects (*P* < 0.001) and older controls (*P* < 0.001) (Figure 4B). However, this was not necessarily accompanied by a reduction in WM capillary length density between PSD and PSND subjects (29) or significant alteration in the % area covered by COL4 immunoreactivity. The % areas with COL4 immunostained capillary profiles were not significantly different between controls, PSND and PSD or VaD groups (*P* > 0.05). Despite the presence of similar burdens of vascular pathology in the PSD and PSND groups, density of capillary pericytes specifically separated poststroke decliners from stable subjects. We also found that fibrinogen reactivity was negatively but weakly correlated with pericyte soma numbers per COL4 area (*r* = −0.3; *P* = 0.057) (Figure 4D).

**Figure 5:**
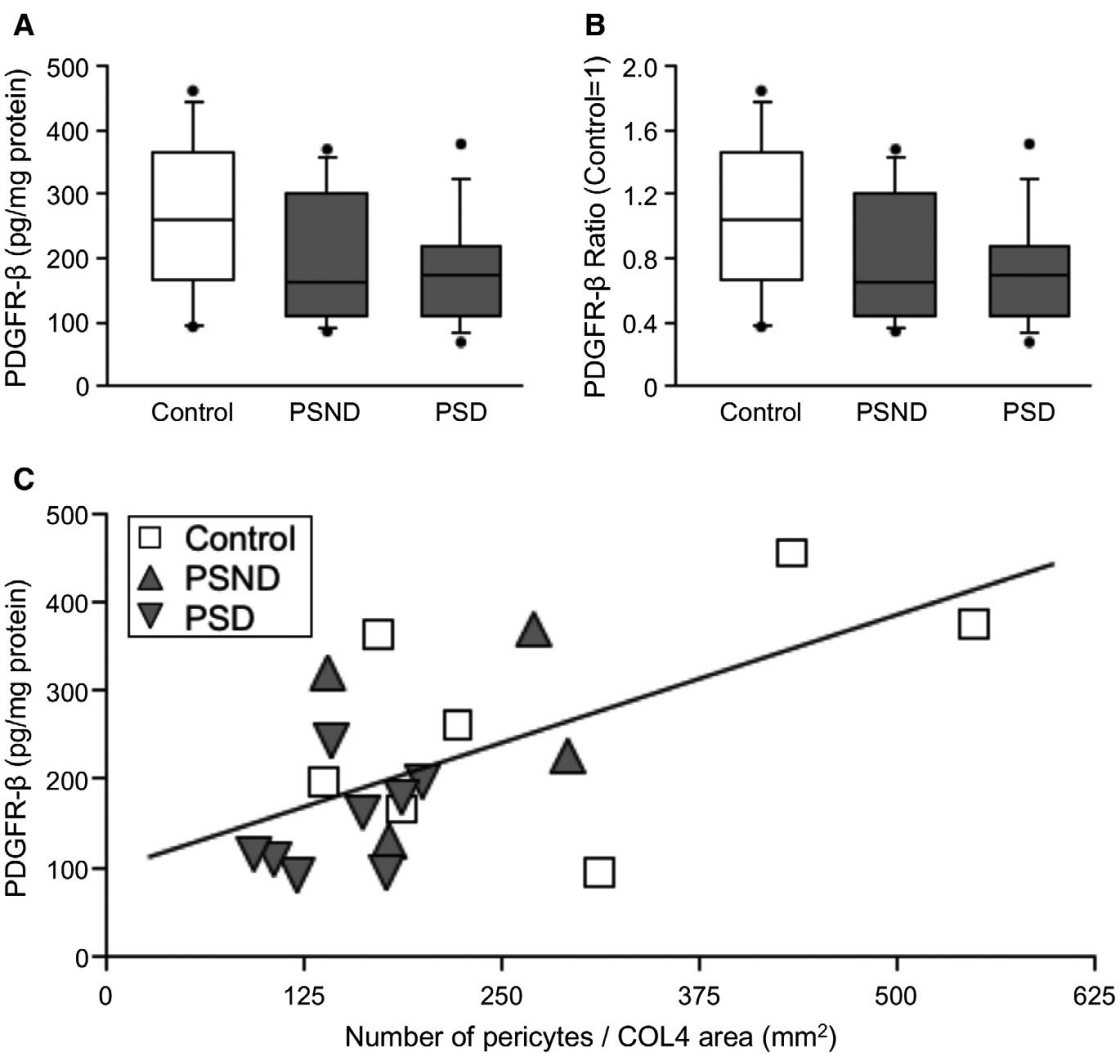
*Quantification of PDGFR‐β reactivity by ELISA in the frontal WM*. **A,B.** Box plots show relative PDGFR‐β reactivity in extracts of the WM from PSD subjects compared to PSND and normal controls. **A.** Plots show PDGFR‐β value (pg/mg protein). **B.** Plots represent PDGFR‐β ratio standardised to control (control = 1). **C.** Correlation of PDGFR‐β reactivity by ELISA and pericyte number per COL4 area in frontal WM. PDGFR‐β detected by ELISA was correlated with pericyte number per COL4 area (Pearson’s *r* = 0.6, *P* = 0.007).

To demonstrate the differential effects in pericytes identified morphologically by COL4 plus the nuclear stain, we next concentrated on comparisons between PSD and PSND subjects. We performed PDGFR‐β immunoassays (ELISA) in available extracts from WM tissue samples adjacent to tissue sections that were used for the morphological analysis. While we found there was a clear trend in PDGFR‐β immunoreactivity in the order: old controls >PSND > PSD (Figure 5A), the mean values for PDGFR‐β assessed by ELISA did not reach statistical significance (*P* > 0.05). PDGFR‐β immunoreactivity was reduced by 30%–40% in PSD and PSND subjects compared to controls (Figure 5B). However, we observed a strong correlation between numbers of pericyte somata and PDGFR‐β reactivities in matched samples (*r* = 0.6, *P* = 0.007) (Figure 5C). These results confirmed the relationship between pericyte cell numbers and PDGFR‐β reactivity in the WM and indicated trends in lower expression of PDGFR‐β in PSD subjects.

**Figure 6:**
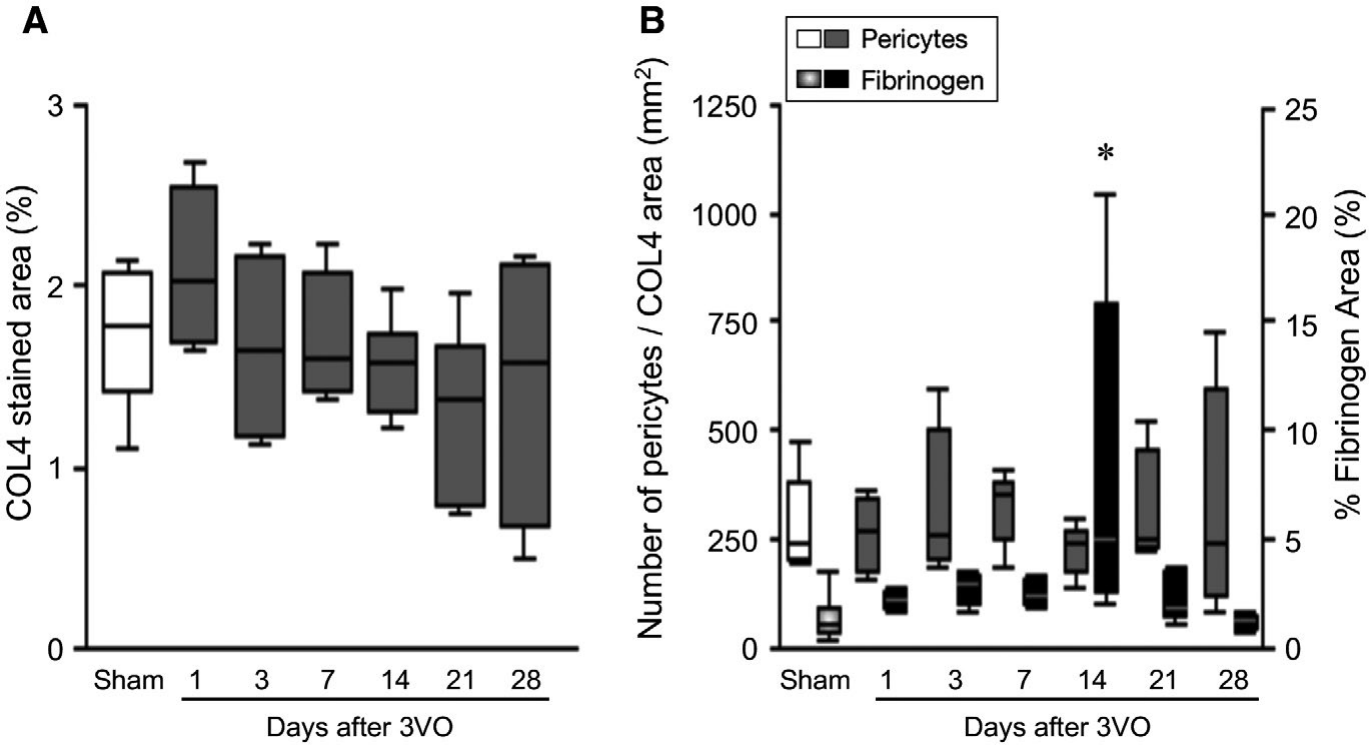
*Integrity of the BBB and pericyte numbers in the frontal WM in a nonhuman primate model of cerebral hypoperfusion*. **A.** Box plots showing mean vascular density (% COL4 stained area) in the frontal WM of adult baboons subjected to 3VO. Each time point represents mean of n = 4–8 animals and the results were computed from both hemispheres. There were no differences between the right and left sides. Microvascular density was not changed after 3VO compared to Sham operated animals (*P* > 0.05). **B.** Quantification of pericytes density and fibrinogen reactivity in the frontal WM of adult baboons subjected to 3VO. There was a high variation in fibrinogen immunoreactivity over survival time (*P* < 0.01). Fibrinogen immunoreactivity was the highest at 14 days after 3VO compared to Sham, 1 and 28 days after 3VO groups (*P < 0.05), concomitant with relatively lower pericyte density at 14 days.

In further experiments, we tested the hypothesis if fibrinogen leakage was specifically associated with changes in pericytes. Using WM tissues from a previously described baboon model of cerebral hypoperfusion, we found that pericytes numbers determined using the same methods above were reduced at 14 days after 3 vessels occlusion (3VO) (Figure 6B), whereas mean vascular density as assessed by COL4 immunoreactivities were not altered over the survival period (Figure 6A). This was concomitant with peak of fibrinogen reactivity in the WM tissues (Figure 6B). All original data presented in this article are available for review.

## Discussion

In this study, we explored actual numbers of pericytes with a nucleus on capillaries rather than coverage of pericyte processes in the deep WM across several dementias with vascular pathology. Using conventional immunohistochemical and immunofluorescence methods, we first demonstrated that pericyte somata are contiguous with COL4 immunostained capillary network and microvascular profiles. We then determined numbers of pericytes with the requirement that each cell soma should contain a nucleus to be considered as a bona fide capillary pericyte. We manually only counted pericyte bodies with clear visible nuclei, which were always located abluminally and counted at high power magnification as typical bumps or protruded bodies into the parenchyma. We are confident these were not mistaken for protruding nucleated endothelial cells because previously in assessing some 0.7 million profiles of capillaries (29), we only observed occasional luminal blebs arising from endothelial cells. Unlike endothelial cells, we also found pericyte cells bodies were GLUT1 negative but positive for COL4 or for PDGFR‐β (15). Furthermore, we noted endothelial cells were few times greater in number than the pericyte cell bodies. This is consistent with greater densities of FLI1 nuclear marker used to track retinal endothelial cells, which were 20–30 per mm capillary length (55). Irrespective, as a caveat it is not unlikely that we have occasionally included endothelial cell nuclei in the manual counts (43). However, overall the current described criteria and our previous observations (15), it is clearly possible to distinguish differences in the morphology and location of nuclei of pericytes and endothelial cells (43).

Consistent with the finding that COL4 is a viable marker to identify pericyte soma, we had previously reported a strong relationship between COL4 and PDGFR‐β immunoreactivities but not GLUT1 and PDGFR‐β in WM (15). Although pericyte numbers were determined using 2D imaging analysis rather than 3D stereology, we are confident that the estimated numbers are close to the actual because in our previous study (11), we did not find substantial differences in capillary length determination using the two quantitative morphological approaches. Our findings indicate that pericyte cell density was approximately 2 per mm capillary length in the WM. This number was slightly higher in the overlying frontal cortex and was estimated to be 3–5 per mm capillary length (22). Given these numbers and knowing the approximate length density of capillaries in the human brain is 650 km (6), we estimate the range of numbers of capillary pericytes at any one time in normal aging human brain is ~0.4–1.0 billion. This may be an over estimate and does not include pericytes and pericyte‐like cells that regulate the circulation in larger vessels including arterioles and penetrating arteries (15, 21). Consistent with these observations, Berthiaume *et al* (8) indicate ~2–3 pericyte nuclei per mm of mid‐capillary region in the mouse cortex. Our study also demonstrates a reliable morphological method to estimate numbers of nucleated pericytes in COL4 or similar markers such as laminin that capture components of the basement membrane. The pericyte cell bodies with a nucleus protected by a visible COL4 positive membrane suggests viable cells that may not necessarily reflect the same as when pericyte coverage is used to determine pericyte status. However, our observations are consistent with previous studies (44). We could not reliably assess pericyte coverage with antibodies to PDGFR‐β in human post‐mortem tissue. We attempted immunostaining of large numbers of sections with PDGFR‐β antibodies, but it was never as consistent or robust as COL4 immunohistochemistry. However, we were able to use immunofluorescence to verify that COL4 stained pericytes are bona fide pericyte soma.

Although an earlier ultrastructural study using neurosurgical biopsies (51) showed that pericyte cell area in capillaries of the WM underlying the frontal and temporal cortex was substantially reduced (>50%) in 80 year olds compared to 20 years olds, we did not detect a significant difference in our younger and older controls. However, the loss of pericytes with age implies decreased ability of the BBB to compensate for transient leaks in the elderly. In the study by Stewart and colleagues (51), however, it was noteworthy pericyte cell area in human brain was 2–3 times lower in the WM compared to the gray matter. By proxy, this corroborates our observations on the differential in pericyte cell nuclei between frontal WM and frontal cortex capillaries (22) (RD, YH, RK unpublished observations).

The most important finding was that capillary pericytes were reduced in the WM in PSD and VaD subjects and those with other common dementias including Mixed and AD. Given significant SVD pathology associated with VaD and Mixed dementia (18), it is not surprising that pericytes were decreased in these old age dementias. However, the remarkable observation was that pericytes were specifically impaired or degenerated in poststroke survivors who developed dementia (PSD) compared those who did not (poststroke no dementia; PSND). This cellular alteration occurred in parallel to fibrinogen leakage and apparent in the deep WM, which is affected by demyelination and SVD pathology in the aging‐associated dementias. Previous studies have suggested that while ablation of a single pericyte soma in rodents does not affect focal BBB function (8), the absence of pericytes induces microvessel leakage and microvessel regression (44, 54). Fibrinogen leakage related toxicity has been implicated in the loss or degeneration of pericytes (40). Our results in the 3VO nonhuman primates, where we could also completely control any post‐mortem artifacts supports such a link between fibrinogen leakage and pericyte degeneration. However, it is not unlikely that there is specific disruption of the gliovascular unit (30) particularly associated with frontal WMH volumes (13). We have shown that this is characterized by widespread astrocytic clasmatodendrosis involving removal of astrocyte end‐feet and aquaporin IV positive water channel protein from the capillary walls in the deep WM of PSD (13) as well as CADASIL patients (27).

Our observations in AD on reduced pericyte numbers in the WM are remarkably consistent with pericyte coverage measured by PDGFR‐β immunoreactivity in WM of the prefrontal cortex (44). They found 50% loss of pericyte coverage associated with accumulation of extravascular fibrinogen in the subcortical WM of AD subjects. They suggested that pericytes instigate WM disease associated with SVD consistent with the dynamic structural remodeling in disease (9). However, our observations are somewhat at odds with the study of Miners *et al* (42) in which PDGFR‐β reactivity was assayed by ELISA. They reported that although there was fibrinogen leakage and reduced oxygenation, as assessed by the myelin‐associated glycoprotein: proteolipid protein‐1 ratio, in the underlying WM of the precuneus in the parietal lobe, PDGFR‐β reactivity was not significantly changed in AD subjects. This may indicate there is a lack of change in actual pericytes in WM of the parietal lobe in AD. Previous studies (42, 47) have further suggested that amyloid β is likely directly toxic to pericytes. Our results are not compatible with the role of soluble or insoluble amyloid β in pericyte degeneration in the frontal WM across the VaDs and AD. In addition, we have previously reported that both PSD and PSND groups had similar amyloid β load (1). Our results argue for a different mechanism in specific pericyte loss associated with WM degeneration that may primarily relate to BBB damage (44). In prior (42, 44) and our studies here, we found that fibrinogen reactivity was increased in the WM indicating degrees of WM BBB damage in different dementias. However, fibrinogen was also increased in PSND as it was in PSD subjects. This was not surprising given that PSND group exhibited high degree of WM pathology. It is plausible that in PSD subjects there is more advanced disruption of other cellular elements together that progressively reaches a certain threshold to disrupt WM fibers and precipitate dementia.

In view of the unavailability of robust markers of pericytes for use in human tissues (50), we propose that COL4 could be readily applied to determine cell densities of microvascular pericytes in disease and in experimental animals. Previous studies have used several markers to label pericytes, such as PDGFR‐β, NG2, BMP4, αSMA, endosialin, angiopoietin‐1 (Ang‐1), RGS5 and desmin (4, 39, 56). However, there is no single marker specific for pericytes; a unique molecular signature of pericytes has not yet been uncovered. PDGFR‐β immunoreactivity is the most used pericyte marker but it is also apparent in neurons, myofibroblasts, fibroblasts, vascular smooth muscle cells and endothelial precursor cells (3, 19). Unlike in cerebral cortex, we found PDGFR‐β immunoreactivity in the human WM to be mostly but not solely associated with WM pericytes. This finding was consistent with the correlation between pericyte numbers per COL4 area in mm^2^ and PDGFR‐β immunoreactivity that also verified that the loss of pericytes in PSD was related to decreased PDGFR‐β. The lower expression of PDGFR‐β immunoreactivity in PSND may be explained by increasing atrophy of pericyte cell processes that had not yet lost their nuclei.

The loss or degradation of pericytes could occur because of hemodynamic changes and greater degree of endothelial damage at the molecular level that progresses in subjects who develop cognitive impairment or dementia (46). We previously found that a noninvasive procedure such as long‐term bilateral carotid artery stenosis causing cerebral hypoperfusion in the WM triggers disruption of the gliovascular unit including severe clasmatodendrosis likely because of hemodynamic changes in the circulation (28). It is not unlikely that a vicious cycle is set up whereby detachment of perivascular cells such as pericytes than are exposed to blood‐derived proteins which are then create a toxic environment on the abluminal side of the capillary network.

Our study has a few limitations. First, we did not assess pericyte numbers in the WM of other lobes of the brain, for example in previously reported regions such as that underlying the precuneus. We surmised that quantification of other regions required a monumental effort that may not reveal different results given that WM changes associated with SVD and possible fibrinogen leakage may have similar outcomes. Second, we used straightforward 2D measurements rather than 3D stereology in the quantification of pericyte numbers. We reasoned that it was unnecessary since we had previously shown that the qualitative changes described using 2D measurements are similar to those obtained with 3D stereology (11), which is cumbersome for large numbers of samples. While further labor‐intensive work could provide reveal precise numbers and turnover of pericytes, we do not believe our estimates of pericyte numbers are grossly over. The pertinent finding here is that capillary pericyte somata were fewer in subjects who developed dementia. Third, the availability of more specific markers of pericytes would also have been useful to verify our findings on the mechanics of pericyte cell impairment or turnover and determine if PDGFR‐β is decreased intracellularly prior to complete degeneration in the persistently hypoxic state within the deep WM in aging‐related dementias (23).

In summary, using a relatively simple morphometric method, we found that numbers of capillary pericytes in the WM were reduced across all common dementias but particularly in individuals who develop dementia after stroke injury. Pericyte cell loss is likely associated with disintegration of the gliovascular unit of the WM that impairs BBB function. These changes in the deep WM are remote from any direct local tissue injury and consistent with the notion that degenerative processes in the WM are instigated by perfusion disturbances from the circulation. Our observations suggest pericytes as components of the gliovascular unit of the WM are an important target for therapeutic interventions.

## Ethical approval

Ethical approvals were granted by local research ethics committees of the Newcastle upon Tyne Foundation Hospitals Trust. Permission for use of brains for post‐mortem research was also granted by consent from next‐of‐kin or family. All the brain tissues were retained in and obtained from the Newcastle Brain Tissue Resource.

## Conflict of interest

The authors have no disclosures or conflicts of interest in relation to this manuscript.

## Author contributions

Ren Ding: providing first draft, most analysis, interpretation and acquisition of data.

Yoshiki Hase: analysis, interpretation, acquisition of data and technical advice on imaging.

Kamar Ameen‐Ali: acquisition of data, technical advice on assays and revising the manuscript.

Michael Ndung’u: acquisition of experimental data and interpretation.

William Stevenson: acquisition and interpretation of data.

Joseph Barsby: acquisition of data and assistance with the assays.

Ryan Gourlay: acquisition of data and assistance with the assays.

Tolulope Akinyemi: acquisition of data and assistance with the assays.

Rufus Akinyemi: revising the manuscript and interpretation of data.

Maiko Uemura: revising the manuscript and interpretation of data.

Tuomo Polvikoski: case diagnosis and acquisition of data.

Elizabeta Mukaetova‐Ladinska: revising manuscript, assistance with assays and data analysis.

Masafumi Ihara: revising the manuscript and interpretation of data.

Raj N. Kalaria: drafting, revising the manuscript and interpretation of data, diagnosing the cases and obtaining funding.

## Data Availability

The data that support the findings of this study are available on request from the corresponding author. The data are not publicly available due to privacy or ethical restrictions.

## References

[1] Akinyemi RO , Allan LM , Oakley A , Kalaria RN (2017) Hippocampal neurodegenerative pathology in post‐stroke dementia compared to other dementias and aging controls. Front Neurosci 11:717. 29311794 10.3389/fnins.2017.00717 PMC5742173

[2] Allan LM , Rowan EN , Firbank MJ , Thomas AJ , Parry SW , Polvikoski TM *et al* (2011) Long term incidence of dementia, predictors of mortality and pathological diagnosis in older stroke survivors. Brain 134(Pt 12):3716–3727. 22171356 10.1093/brain/awr273 PMC3235558

[3] Andrae J , Gallini R , Betsholtz C (2008) Role of platelet‐derived growth factors in physiology and medicine. Genes Dev 22:1276–1312. 18483217 10.1101/gad.1653708 PMC2732412

[4] Armulik A , Genove G , Betsholtz C (2011) Pericytes: developmental, physiological, and pathological perspectives, problems, and promises. Dev Cell 21:193–215. 21839917 10.1016/j.devcel.2011.07.001

[5] Baradaran H , Mtui EE , Richardson JE , Delgado D , Dunning A , Marshall RS *et al* (2016) White matter diffusion abnormalities in carotid artery disease: a systematic review and meta‐analysis. J Neuroimaging 26:481–488. 27079165 10.1111/jon.12347

[6] Begley DJ , Brightman MW (2003) Structural and functional aspects of the blood‐brain barrier. Prog Drug Res 61:39–78. 14674608 10.1007/978-3-0348-8049-7_2

[7] Bell RD , Winkler EA , Sagare AP , Singh I , LaRue B , Deane R , Zlokovic BV (2010) Pericytes control key neurovascular functions and neuronal phenotype in the adult brain and during brain aging. Neuron 68:409–427. 21040844 10.1016/j.neuron.2010.09.043 PMC3056408

[8] Berthiaume AA , Grant RI , McDowell KP , Underly RG , Hartmann DA , Levy M *et al* (2018) Dynamic remodeling of pericytes in vivo maintains capillary coverage in the adult mouse brain. Cell Rep 22:8–16. 29298435 10.1016/j.celrep.2017.12.016 PMC5782812

[9] Berthiaume AA , Hartmann DA , Majesky MW , Bhat NR , Shih AY (2018) Pericyte structural remodeling in cerebrovascular health and homeostasis. Front Aging Neurosci 10:210. 30065645 10.3389/fnagi.2018.00210 PMC6057109

[10] Betsholtz C , Keller A (2014) PDGF, pericytes and the pathogenesis of idiopathic basal ganglia calcification (IBGC). Brain Pathol 24:387–395. 24946076 10.1111/bpa.12158 PMC8029277

[11] Burke MJ , Nelson L , Slade JY , Oakley AE , Khundakar AA , Kalaria RN (2014) Morphometry of the hippocampal microvasculature in post‐stroke and age‐related dementias. Neuropathol Appl Neurobiol 40:284–295. 24003901 10.1111/nan.12085 PMC4282329

[12] Carmeliet P , Jain RK (2011) Molecular mechanisms and clinical applications of angiogenesis. Nature 473:298–307. 21593862 10.1038/nature10144 PMC4049445

[13] Chen A , Akinyemi RO , Hase Y , Firbank MJ , Ndung'u MN , Foster V *et al* (2016) Frontal white matter hyperintensities, clasmatodendrosis and gliovascular abnormalities in ageing and post‐stroke dementia. Brain 139(Pt 1):242–258. 26667280 10.1093/brain/awv328 PMC4905522

[14] Chen A , Oakley AE , Monteiro M , Tuomela K , Allan LM , Mukaetova‐Ladinska EB *et al* (2016) Multiplex analyte assays to characterize different dementias: brain inflammatory cytokines in poststroke and other dementias. Neurobiol Aging 38:56–67. 26827643 10.1016/j.neurobiolaging.2015.10.021 PMC4759608

[15] Craggs LJ , Fenwick R , Oakley AE , Ihara M , Kalaria RN (2015) Immunolocalization of platelet‐derived growth factor receptor‐beta (PDGFR‐beta) and pericytes in cerebral autosomal dominant arteriopathy with subcortical infarcts and leukoencephalopathy (CADASIL). Neuropathol Appl Neurobiol 41:557–570. 25303037 10.1111/nan.12188 PMC5098250

[16] Craggs LJ , Hagel C , Kuhlenbaeumer G , Borjesson‐Hanson A , Andersen O , Viitanen M *et al* (2013) Quantitative vascular pathology and phenotyping familial and sporadic cerebral small vessel diseases. Brain Pathol 23:547–557. 23387519 10.1111/bpa.12041 PMC8029230

[17] Debette S , Markus HS (2010) The clinical importance of white matter hyperintensities on brain magnetic resonance imaging: systematic review and meta‐analysis. BMJ 341:c3666. 20660506 10.1136/bmj.c3666 PMC2910261

[18] Deramecourt V , Slade JY , Oakley AE , Perry RH , Ince PG , Maurage CA , Kalaria RN (2012) Staging and natural history of cerebrovascular pathology in dementia. Neurology 78:1043–1050. 22377814 10.1212/WNL.0b013e31824e8e7f PMC3317531

[19] Dias Moura Prazeres PH , Sena IFG , Borges IDT , de Azevedo PO , Andreotti JP , de Paiva AE *et al* (2017) Pericytes are heterogeneous in their origin within the same tissue. Dev Biol 427:6–11. 28479340 10.1016/j.ydbio.2017.05.001 PMC6076854

[20] Diaz‐Flores L , Gutierrez R , Madrid JF , Varela H , Valladares F , Acosta E *et al* (2009) Pericytes. Morphofunction, interactions and pathology in a quiescent and activated mesenchymal cell niche. Histol Histopathol 24:909–969. 19475537 10.14670/HH-24.909

[21] Diaz‐Flores L , Gutierrez R , Varela H , Rancel N , Valladares F (1991) Microvascular pericytes: a review of their morphological and functional characteristics. Histol Histopathol 6:269–286. 1802127

[22] Ding R , Barsby J , Slade J , Oakley A , Kalaria R (2017) The role of cerebral pericytes in the frontal white matter of ageing related dementias. Alzheimers Dement 13:1483–1484.

[23] Fernando MS , Simpson JE , Matthews F , Brayne C , Lewis CE , Barber R *et al* (2006) White matter lesions in an unselected cohort of the elderly: molecular pathology suggests origin from chronic hypoperfusion injury. Stroke 37:1391–1398. 16627790 10.1161/01.STR.0000221308.94473.14

[24] Gouw AA , Seewann A , Vrenken H , van der Flier WM , Rozemuller JM , Barkhof F *et al* (2008) Heterogeneity of white matter hyperintensities in Alzheimer's disease: post‐mortem quantitative MRI and neuropathology. Brain 131(Pt 12):3286–3298. 18927145 10.1093/brain/awn265

[25] Graham C , Santiago‐Mugica E , Abdel‐All Z , Li M , McNally R , Kalaria RN , Mukaetova‐Ladinska EB (2019) Erythrocytes as biomarkers for dementia: analysis of protein content and alpha‐synuclein. J Alzheimers Dis 71:569–580. 31424413 10.3233/JAD-190567

[26] Hall CN , Reynell C , Gesslein B , Hamilton NB , Mishra A , Sutherland BA *et al* (2014) Capillary pericytes regulate cerebral blood flow in health and disease. Nature 508:55–60. 24670647 10.1038/nature13165 PMC3976267

[27] Hase Y , Chen A , Bates LL , Craggs LJL , Yamamoto Y , Gemmell E *et al* (2018) Severe white matter astrocytopathy in CADASIL. Brain Pathol 28:832–843. 29757481 10.1111/bpa.12621 PMC8028291

[28] Hase Y , Craggs L , Hase M , Stevenson W , Slade J , Chen A *et al* (2018) The effects of environmental enrichment on white matter pathology in a mouse model of chronic cerebral hypoperfusion. J Cereb Blood Flow Metab 38:151–165. 28273725 10.1177/0271678X17694904 PMC5757440

[29] Hase Y , Ding R , Harrison G , Hawthorne E , King A , Gettings S *et al* (2019) White matter capillaries in vascular and neurodegenerative dementias. Acta Neuropathol Commun 7:16. 30732655 10.1186/s40478-019-0666-x PMC6366070

[30] Hase Y , Horsburgh K , Ihara M , Kalaria RN (2018) White matter degeneration in vascular and other ageing‐related dementias. J Neurochem 144:617–633. 29210074 10.1111/jnc.14271

[31] Hase Y , Polvikoski TM , Firbank MJ , Craggs LJL , Hawthorne E , Platten C *et al* (2019) Small vessel disease pathological changes in neurodegenerative and vascular dementias concomitant with autonomic dysfunction. Brain Pathol 30:191–202. 31357238 10.1111/bpa.12769 PMC8018165

[32] Hase Y , Polvikoski TM , Ihara M , Hase M , Zafar R , Stevenson W *et al* (2019) Carotid artery disease in post‐stroke survivors and effects of enriched environment on stroke pathology in a mouse model of carotid artery stenosis. Neuropathol Appl Neurobiol 45:681–697. 30947376 10.1111/nan.12550

[33] Ihara M , Polvikoski TM , Hall R , Slade JY , Perry RH , Oakley AE *et al* (2010) Quantification of myelin loss in frontal lobe white matter in vascular dementia, Alzheimer's disease, and dementia with Lewy bodies. Acta Neuropathol 119:579–589. 20091409 10.1007/s00401-009-0635-8 PMC2849937

[34] Inzitari D , Pracucci G , Poggesi A , Carlucci G , Barkhof F , Chabriat H *et al* (2009) Changes in white matter as determinant of global functional decline in older independent outpatients: three year follow‐up of LADIS (leukoaraiosis and disability) study cohort. BMJ 339:b2477. 19581317 10.1136/bmj.b2477 PMC2714680

[35] Kalaria RN (2012) Cerebrovascular disease and mechanisms of cognitive impairment: evidence from clinicopathological studies in humans. Stroke 43:2526–2534. 22879100 10.1161/STROKEAHA.112.655803

[36] Kalaria RN (2016) Neuropathological diagnosis of vascular cognitive impairment and vascular dementia with implications for Alzheimer's disease. Acta Neuropathol 131:659–685. 27062261 10.1007/s00401-016-1571-z PMC4835512

[37] Kalaria RN , Kenny RA , Ballard CG , Perry R , Ince P , Polvikoski T (2004) Towards defining the neuropathological substrates of vascular dementia. J Neurol Sci 226:75–80. 15537525 10.1016/j.jns.2004.09.019

[38] Kamouchi M , Ago T , Kitazono T (2011) Brain pericytes: emerging concepts and functional roles in brain homeostasis. Cell Mol Neurobiol 31:175–193. 21061157 10.1007/s10571-010-9605-x PMC11498428

[39] Krueger M , Bechmann I (2010) CNS pericytes: concepts, misconceptions, and a way out. Glia 58:1–10. 19533601 10.1002/glia.20898

[40] Lowe J , Kalaria RN (2015) Chapter 50: Dementia. In: Greenfield's Neuropathology, S Love , P Arie , J Ironside , H Budka (eds), pp. 1001–1055. CRC Press: London.

[41] Matthews FE , Brayne C , Lowe J , McKeith I , Wharton SB , Ince P (2009) Epidemiological pathology of dementia: attributable‐risks at death in the Medical Research Council Cognitive Function and Ageing Study. PLoS Med 6:e1000180. 19901977 10.1371/journal.pmed.1000180 PMC2765638

[42] Miners JS , Schulz I , Love S (2018) Differing associations between Abeta accumulation, hypoperfusion, blood‐brain barrier dysfunction and loss of PDGFRB pericyte marker in the precuneus and parietal white matter in Alzheimer's disease. J Cereb Blood Flow Metab 38:103–115. 28151041 10.1177/0271678X17690761 PMC5757436

[43] Mishra A , O'Farrell FM , Reynell C , Hamilton NB , Hall CN , Attwell D (2014) Imaging pericytes and capillary diameter in brain slices and isolated retinae. Nat Protoc 9:323–336. 24434801 10.1038/nprot.2014.019

[44] Montagne A , Nikolakopoulou AM , Zhao Z , Sagare AP , Si G , Lazic D *et al* (2018) Pericyte degeneration causes white matter dysfunction in the mouse central nervous system. Nat Med 24:326–337. 29400711 10.1038/nm.4482 PMC5840035

[45] Montine TJ , Phelps CH , Beach TG , Bigio EH , Cairns NJ , Dickson DW *et al* (2012) National Institute on Aging‐Alzheimer's Association guidelines for the neuropathologic assessment of Alzheimer's disease: a practical approach. Acta Neuropathol 123:1–11. 22101365 10.1007/s00401-011-0910-3 PMC3268003

[46] Nation DA , Sweeney MD , Montagne A , Sagare AP , D'Orazio LM , Pachicano M *et al* (2019) Blood‐brain barrier breakdown is an early biomarker of human cognitive dysfunction. Nat Med 25:270–276. 30643288 10.1038/s41591-018-0297-y PMC6367058

[47] Nortley R , Korte N , Izquierdo P , Hirunpattarasilp C , Mishra A , Jaunmuktane Z *et al* (2019) Amyloid beta oligomers constrict human capillaries in Alzheimer's disease via signaling to pericytes. Science 365:eaav9518. 31221773 10.1126/science.aav9518. PMC6658218

[48] Perry RH , Oakley AE (1993) Newcastle brain map. In: Neuropsychiatric Disorders, GW Roberts , N Leigh , DR Weinberger (eds), pp. 1–10. Wolfe: London.

[49] Skrobot OA , Attems J , Esiri M , Hortobagyi T , Ironside JW , Kalaria RN *et al* (2016) Vascular cognitive impairment neuropathology guidelines (VCING): the contribution of cerebrovascular pathology to cognitive impairment. Brain 139:2957–2969. 27591113 10.1093/brain/aww214

[50] Smyth LCD , Rustenhoven J , Scotter EL , Schweder P , Faull RLM , Park TIH , Dragunow M (2018) Markers for human brain pericytes and smooth muscle cells. J Chem Neuroanat 92:48–60. 29885791 10.1016/j.jchemneu.2018.06.001

[51] Stewart PA , Magliocco M , Hayakawa K , Farrell CL , Del Maestro RF , Girvin J *et al* (1987) A quantitative analysis of blood‐brain barrier ultrastructure in the aging human. Microvasc Res 33:270–282. 3587079 10.1016/0026-2862(87)90022-7

[52] Uemura MT , Ihara M , Maki T , Nakagomi T , Kaji S , Uemura K *et al* (2018) Pericyte‐derived bone morphogenetic protein 4 underlies white matter damage after chronic hypoperfusion. Brain Pathol 28:521–535. 28470822 10.1111/bpa.12523 PMC6099372

[53] Uemura MT , Maki T , Ihara M , Lee VMY , Trojanowski JQ (2020) Brain microvascular pericytes in vascular cognitive impairment and dementia. Front Aging Neurosci 12:80. 32317958 10.3389/fnagi.2020.00080 PMC7171590

[54] von Tell D , Armulik A , Betsholtz C (2006) Pericytes and vascular stability. Exp Cell Res 312:623–629. 16303125 10.1016/j.yexcr.2005.10.019

[55] Watson EC , Koenig MN , Grant ZL , Whitehead L , Trounson E , Dewson G , Coultas L (2016) Apoptosis regulates endothelial cell number and capillary vessel diameter but not vessel regression during retinal angiogenesis. Development 143:2973–2982. 27471260 10.1242/dev.137513

[56] Winkler EA , Bell RD , Zlokovic BV (2011) Central nervous system pericytes in health and disease. Nat Neurosci 14:1398–1405. 22030551 10.1038/nn.2946 PMC4020628

[57] Yamamoto Y , Craggs LJ , Watanabe A , Booth T , Attems J , Low RW *et al* (2013) Brain microvascular accumulation and distribution of the NOTCH3 ectodomain and granular osmiophilic material in CADASIL. J Neuropathol Exp Neurol 72:416–431. 23584202 10.1097/NEN.0b013e31829020b5

